# SIRT1 regulates nuclear number and domain size in skeletal muscle fibers

**DOI:** 10.1002/jcp.26542

**Published:** 2018-03-25

**Authors:** Jacob A. Ross, Yotam Levy, Kristoffer Svensson, Andrew Philp, Simon Schenk, Julien Ochala

**Affiliations:** ^1^ School of Basic and Medical Biosciences Faculty of Life Sciences and Medicine King's College London London UK; ^2^ Department of Orthopaedic Surgery University of California San Diego La Jolla California; ^3^ Biomedical Sciences Graduate Program University of California San Diego La Jolla California; ^4^ School of Sport and Exercise Sciences University of Birmingham Birmingham UK

**Keywords:** force production, myofiber, myonuclei, PGC‐1α, SIRT1

## Abstract

Skeletal muscle fibers are giant multinucleated cells wherein individual nuclei govern the protein synthesis in a finite volume of cytoplasm; this is termed the myonuclear domain (MND). The factors that control MND size remain to be defined. In the present study, we studied the contribution of the NAD^+^‐dependent deacetylase, sirtuin 1 (SIRT1), to the regulation of nuclear number and MND size. For this, we isolated myofibers from mice with tissue‐specific inactivation (mKO) or inducible overexpression (imOX) of SIRT1 and analyzed the 3D organisation of myonuclei. In imOX mice, the number of nuclei was increased whilst the average MND size was decreased as compared to littermate controls. Our findings were the opposite in mKO mice. Muscle stem cell (satellite cell) numbers were reduced in mKO muscles, a possible explanation for the lower density of myonuclei in these mice; however, no change was observed in imOX mice, suggesting that other factors might also be involved, such as the functional regulation of stem cells/muscle precursors. Interestingly, however, the changes in the MND volume did not impact the force‐generating capacity of muscle fibers. Taken together, our results demonstrate that SIRT1 is a key regulator of MND sizes, although the underlying molecular mechanisms and the cause‐effect relationship between MND and muscle function remain to be fully defined.

## Graphical abstract

Overexpression of SIRT1 increases myonuclear density and thus reduces myonuclear domain sizes; conversely, ablation of SIRT1 decreases myonuclear density and thus increases myonuclear domain volumes. Altered satellite cell numbers might explain such modulation of myonuclear density within muscle fibers.

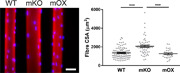

## 1 INTRODUCTION

Skeletal muscle fibers are large cells containing hundreds of nuclei, with each nucleus hypothesised to control the gene transcription and protein synthesis in a finite volume of cytoplasm, which is termed the myonuclear domain (MND) (Edgerton & Roy, 1991; Hall & Ralston, 1989; Ralston & Hall, 1992). Regular spacing between nuclei and optimal MND sizes are required to facilitate transcriptional regulation (Volk, 2013) and minimise the transport distances of gene products (Bruusgaard, Liestol, Ekmark, Kollstad, & Gundersen, 2003). The molecular signaling pathways that determine nuclear positioning and subsequent MND sizes remain unclear.

Recently, we have demonstrated that the transcriptional coactivator, peroxisome proliferator‐activated receptor‐γ coactivator 1‐α (PGC‐1α), plays a key role in regulating the volume of MNDs in myofibers. Indeed, despite the absence of noticeable changes when PGC‐1α is specifically inactivated in muscles, its overexpression induces a dramatic increase in the number of myonuclei and thus a decrease in the MND volume (Ross et al., 2016). As PGC‐1α is a central regulator of mitochondrial biogenesis, this alteration is thought to increase transcriptional capacity for the de novo synthesis of bioenergetic proteins (Ross et al., 2016). Sirtuin 1 (SIRT1) is a class III NAD^+^‐dependant deacetylase, which targets and modulates the activity of various proteins, and in particular is a pivotal mediator of PGC‐1α activity (Gerhart‐Hines et al., 2007; Gurd, Yoshida, Lally, Holloway, & Bonen, 2009); thus we aimed to determine whether directly modifying SIRT1 activity would impact nuclear number/positioning and MND sizes. For this, we used mice with either a skeletal muscle‐specific inactivation (mKO) or inducible overexpression (imOX) of SIRT1.

We found that overexpression of SIRT1 resulted in increased myonuclear density and thus reduced MND sizes; conversely, ablation of SIRT1 resulted in reduced myonuclear density and thus increased MND sizes. These results mirror those for PGC‐1α, which is activated downstream of SIRT1. Altered satellite cell numbers might explain the modulation of myonuclear density within muscle fibers, although differences were only observed in mKO mice, suggesting that other factors such as stem cell fate/regulation might also be involved, and SIRT1 is known to regulate proliferation of muscle precursor cells (Rathbone, Booth, & Lees, 2009). These results implicate SIRT1 in MND size regulation, although the precise mechanisms remain to be determined.

## 2 MATERIALS AND METHODS

### 2.1 Animals

SIRT1 muscle‐specific overexpressing mice (imOX) and SIRT1 muscle‐specific knockout mice (mKO) were generated as previously described (Philp et al., 2011; Schenk et al., 2011; Svensson, LaBarge, Martins, & Schenk, 2017). The transgene in mKO mice was driven by the muscle‐specific muscle creatine kinase (MCK) promoter, while the transgene in imOX mice was driven by the human α‐skeletal actin promoter in a tamoxifen (TMX)‐inducible manner (∼50‐fold increase in SIRT1 protein level). For simplicity, wild‐type (WT) mice refer to littermate controls possessing the LoxP‐flanked SIRT1 transgenes, but with no muscle‐specific Cre. Three imOX, five mKO, and eight WT mice were euthanised at 4 months of age, which was 4 weeks after TMX administration in imOX mice; all littermate controls for imOX mice also received TMX. All experiments were approved and conducted in accordance with the Animal Care Program at UCSD.

### 2.2 Solutions

Relaxing and activating solutions contained 4 mM Mg‐ATP, 1 mM free Mg^2+^, 20 mM imidazole, 7 mM EGTA, 14.5 mM creatine phosphate, and KCl to adjust the ionic strength to 180 mM and pH to 7.0. The concentrations of free Ca^2+^ were 10^−9.00^ M (relaxing solution) and 10^−4.50^ M (activating solution).

### 2.3 Myofiber permeabilization

After dissection, the tibialis anterior (TA) muscle was treated with skinning solution (relaxing solution containing glycerol; 50:50 v/v) for 24 hr at 4 °C, after which they were transferred to −20 °C (Frontera & Larsson, 1997).

### 2.4 Myonuclear organization of single myofibers

On the day of experiment (within 4 weeks of muscle dissection), single myofibers were randomly isolated. Arrays of approximately nine myofibers were prepared at room temperature (RT). For each myofiber, both ends were clamped to half‐split copper meshes designed for electron microscopy (SPI G100 2010C‐XA, width, 3 mm), which had been glued to cover slips (Menzel–Gläser, 22 × 50 mm, thickness 0.13–0.16 mm). For the measurement of nuclear coordinates, fibers were mounted at a fixed sarcomere length of ≈2.20 μm. This was a prerequisite for exact determination of nuclei spatial organisation as it allowed accurate comparisons between myofibers (Ross et al., 2016).

Arrays were subsequently fixed in 4% PFA/PBS, and further permeabilized with 0.1% Triton‐X100/PBS for 10 min each. fibers were then subjected to actin staining (Rhodamine‐conjugated Phalloidin, Molecular Probes, Eugene, OR, R415, 1:100 in PBS) and nuclear staining (DAPI, Molecular Probes, D3571, 1:1000 in PBS). Images were acquired using a confocal microscope (Zeiss Axiovert 200, Cambridge, UK, objective ×20) equipped with CARV II confocal imager (BD Bioscience, San Jose, CA). To visualize muscle fibers in 3D, stacks of 100 images were acquired (1 μm *z* increments) and analyzed with a custom‐made Matlab programme. A distribution score (“g”) is a measure of the regularity of spacing between nuclei; this was calculated as described before (Bruusgaard et al., 2003). Briefly, a theoretical optimum distribution (*M*_O_) and a theoretical random distribution (*M*_R_) were generated for each fiber, based on the numbers and coordinates of the corresponding myonuclei. This was compared with the experimental distribution (*M*_E_), and a “g” score was calculated with the equation: *g* = (*M*_E_−*M*_R_)/(*M*_O_−*M*_R_).

### 2.5 Single muscle fiber force production

Single muscle fibers were dissected following the same procedure as above. They were then individually attached between connectors leading to a force transducer (model 400A; Aurora Scientific, Aurora, ON) and a lever arm system (model 308B; Aurora Scientific). Sarcomere length was set to ≈2.50 μm (known to be optimal for force measurement) and the temperature to 15 °C (Lindqvist, Cheng, Renaud, Hardeman, & Ochala, 2013; Ochala, Iwamoto, Ravenscroft, Laing, & Nowak, 2013; Ochala, Ravenscroft, Laing, & Nowak, 2012). Fiber cross‐sectional area (CSA) was estimated under a brightfield microscope using the fiber width and depth, and assuming an elliptical circumference (Lindqvist et al., 2013; Ochala et al., 2013, 2012). The absolute maximal isometric force generation was calculated as the difference between the total tension in the activating solution (pCa 4.50) and the resting tension measured in the same fiber while in the relaxing solution. Specific force was defined as absolute force divided by CSA.

### 2.6 Immunohistochemistry of muscle cryosections

Ten µm thick cryosections of TA muscle were cut in transverse orientation, air‐dried on slides and stored at −80 °C. On the day of staining, slides were thawed, dried for 10 min and rehydrated in PBS for 10 min.

For satellite cell staining: sections were then fixed in 4% PFA/PBS, and permeabilised with 0.5% Triton‐X100/PBS for 10 min each. After washing in PBS, sections were incubated for 1 hr in mouse‐on‐mouse block/PBS (Vector Laboratories, Peterborough, UK, MKB‐2213), followed by blocking for 1 hr in 10% goat serum/PBS. Antibodies to Pax7 (mouse IgG1 isoform, Developmental Studies Hybridoma Bank, supernatant) and laminin (rabbit IgG, Dako, Z0097) were applied overnight in 10% goat serum/PBS at dilutions of 1:10 and 1:500 respectively (temperature 4 °C). After washes, species and isotype specific secondary antibodies conjugated to Alexa 488 or 594 (Molecular Probes) were applied for 1 hr in 10% goat serum/PBS, with the addition of DAPI (all at a dilution of 1:1000). After washing, slides were mounted in Fluoromount (Southern Biotech, Birmingham, AL).

For fiber type/myosin isoform staining: after rehydration as above, sections were blocked in 1% bovine serum albumin/PBS for 10 min. Antibodies directed against different myosin isoforms were then applied in blocking buffer at dilutions of 1:50, overnight at room temperature: mouse anti‐myosin slow/type I (IgG2b isoform, clone BA‐F8); mouse anti‐myosin fast/type IIa (IgG1 isoform, clone SC‐71); mouse anti‐myosin fast/type IIx (IgM isoform, clone 6H1); mouse anti‐myosin fast/type IIb (IgM isoform, clone BF‐F3). In addition, an antibody recognizing all fast/type II isoforms was utilized (mouse IgG1 isoform, clone MY‐32, Sigma–Aldrich, St. Louis, MO, dilution 1:200). Sections were then washed in PBS/0.025% Tween‐20, and incubated with isotype specific secondary antibodies conjugated to Alexa 488 or 594 in blocking buffer (dilution 1:500) for 1 hr. After washing in PBS, slides were mounted in Fluoromount. BA‐F8, SC‐71, and BF‐F3 were gifts from Dr. Stefano Schiaffino (University of Padua, Italy). 6H1 was a gift from Dr. Joseph Hoh (University of Sydney, Australia).

### 2.7 Statistical analysis

For all the parameters, no significant differences were observed between WT mice corresponding to the mKO or imOX groups, so all WT animals were pooled for data analysis. Additionally, within each group (WT, mKO, or imOX), no differences were found between animals, so all the individual data were pooled together. Data are presented as mean ± standard error of the mean (SEM). One‐way ANOVA with Tukey post‐correction was used to test for significance (*p *< 0.05).

## 3 RESULTS

**Figure 1:**
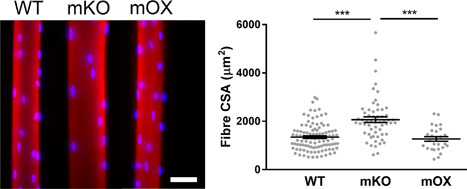
Typical muscle fibers from WT, mKO, and imOX mice. Single muscle fibers were isolated from the tibialis anterior of age‐matched mKO (SIRT1 muscle‐specific knockout), imOX (SIRT1 muscle‐specific overexpression), and WT (wild‐type) mice. These were then stained for actin (Rhodamine Phalloidin; red) and myonuclei (DAPI, blue). Scale bar: 50 μm. Fiber cross‐sectional areas are shown in the graph (each data point represents one fiber; line and error bars are mean ± SEM)

**Figure 2:**
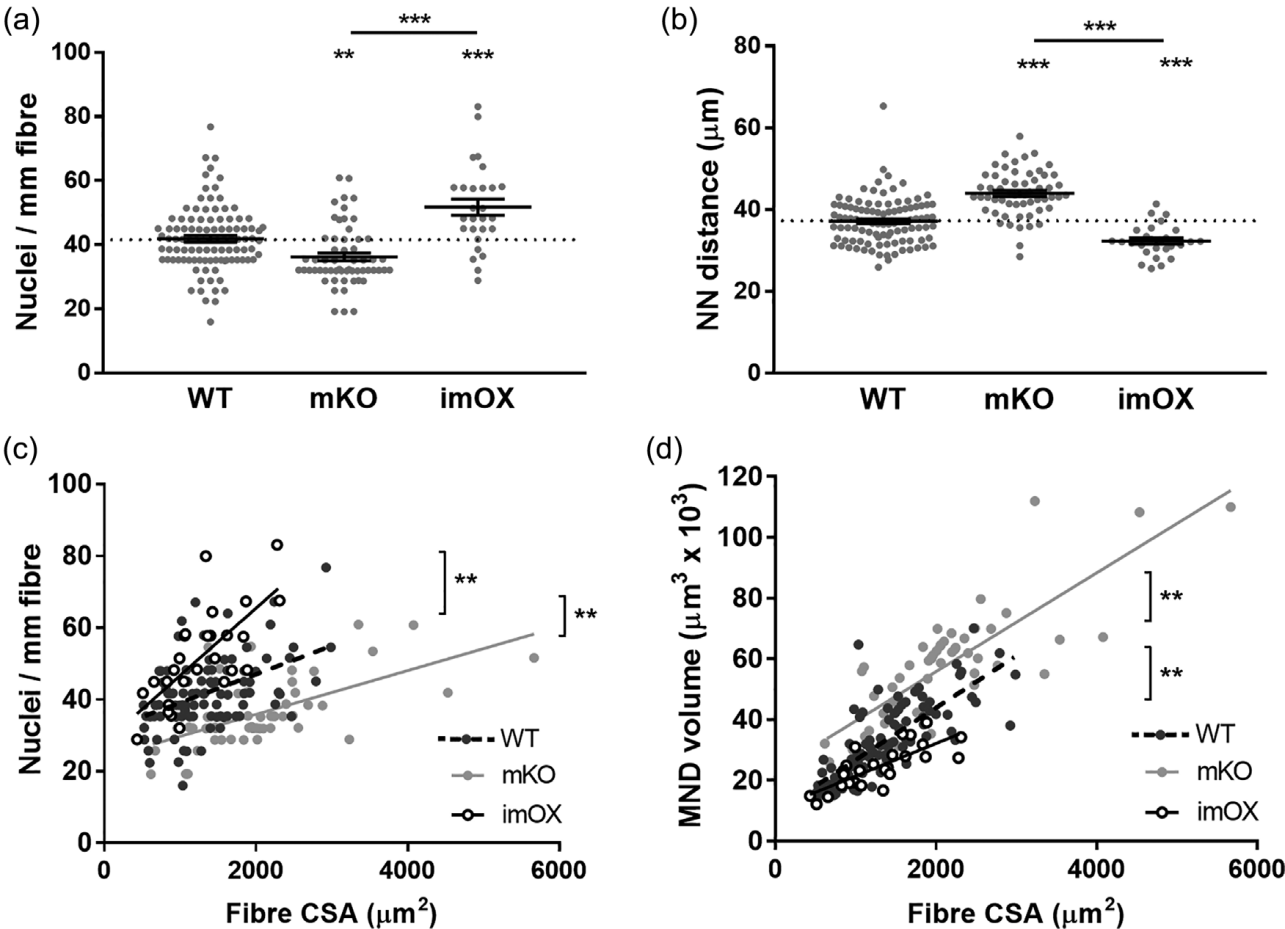
Myonuclear number and domain sizes. MND = myonuclear domain, CSA = fiber cross‐sectional area, NN = nearest neighbour. (a) number of nuclei per mm of fiber length; (b) Nearest neighbour distances between each myonucleus; (c) scatter plot/correlation of nuclei per mm length of fiber and CSA; (d) scatter plot/correlation of MND volume and CSA. Each data point represents one fiber; line and error bars are mean ± SEM. Asterisks directly above data columns denote significance versus WT; Asterisks with a line/bracket denote significance between two other groups. All regression lines demonstrated a statistically significant correlation (*p* < 0.05). In addition, linear regression analysis showed that the elevations between each data line were significantly different from each other (*p* < 0.01 in each case; brackets and asterisks denote differences between lines)

We isolated individual muscle fibers from imOX (*n* = 27), mKO (*n* = 55), and WT mice (*n* = 103). We then membrane‐permeabilised the myofibers, generated confocal microscope reconstructions, and evaluated the 3D spatial arrangement of myonuclei (Figure 1) (Ross et al., 2016). Fiber CSA was similar between WT and imOX, but significantly larger in mKO mice compared to the other groups. We observed that the number of nuclei per mm fiber length was significantly smaller in mKO than WT, and larger in imOX than WT (Figure 2a).

As shown previously (Ross et al., 2016), the number of nuclei per mm fiber length and the average MND size for a particular fiber was related to its CSA with a positive, linear correlation; this was the case in all groups (Figures 2c and 2d; in panel C, *R* ^2^ values for WT, mKO, and imOX were 0.20, 0.34, and 0.50 respectively; in panel D, *R* ^2^ values were 0.59, 0.64, and 0.58 respectively). Additionally, there was a significant difference between elevations of the regression lines of both mKO and imOX compared to WT mice, indicating that: (1) across all fiber sizes, there were more nuclei per mm fiber length in imOX and fewer in mKO compared to WT rodents; and that (2), in accordance with this, MND sizes were larger in mKO and smaller in imOX compared to WT rodents (Figures 2c and 2d). MND sizes are positively correlated with average nearest neighbour (NN) distances between nuclei, and therefore these values were significantly greater in mKO than WT, and significantly smaller in imOX than WT (Figure 2b).

**Figure 3:**
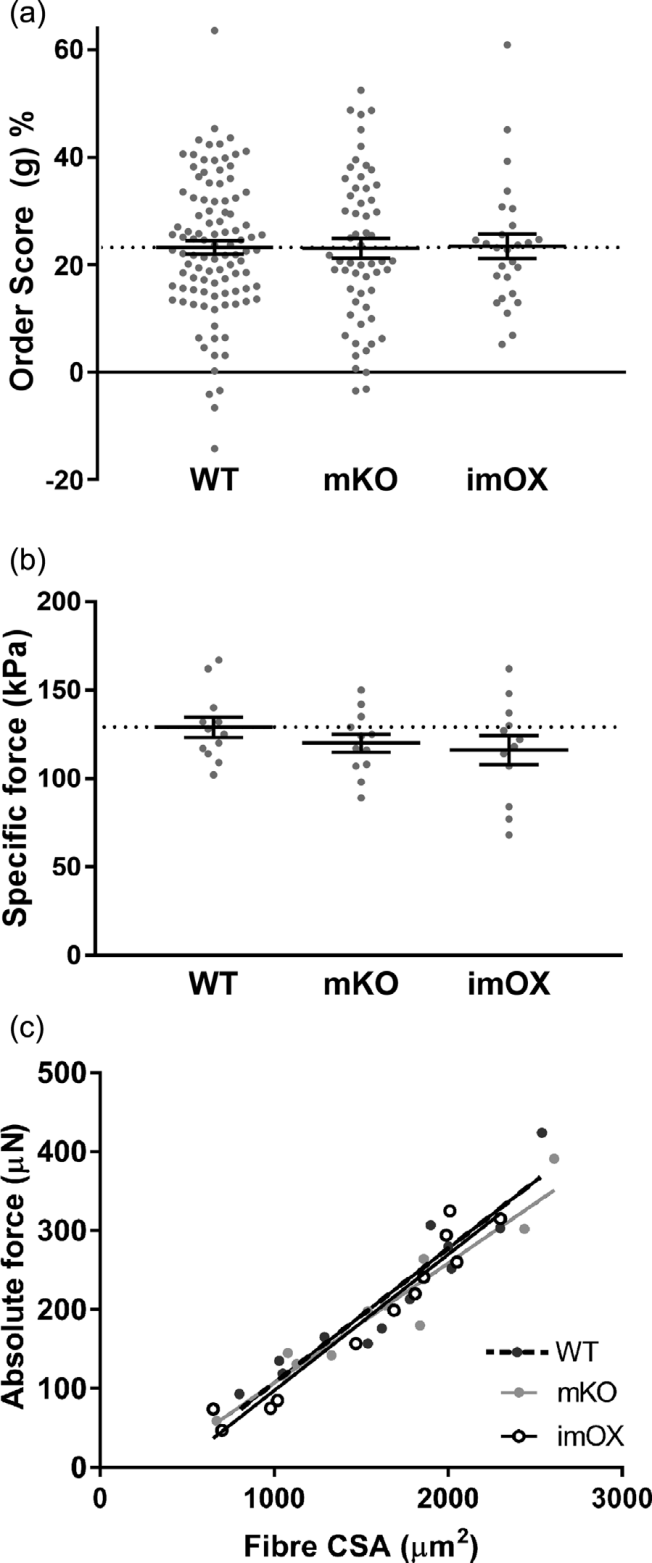
Myonuclear positioning and intrinsic force production in myofibers. (a) Order score calculated from nearest neighbour distances between myonuclei of each fiber (a higher order score indicates more even/regular spacing between nuclei). (b) Specific force production (i.e., absolute force normalised to fiber cross sectional area, CSA). (c) Scatter plot/correlation between CSA and absolute force. Each data point represents one fiber; line and error bars are mean ± SEM

The MND measurements provide valuable information on the average volume controlled by each myonucleus; however, they do not allow characterisation of the spatial arrangement of myonuclei within the whole fiber. Therefore, to evaluate the regularity of nuclear positioning within myofibers, a distribution score (“g”) was evaluated (Bruusgaard et al., 2003), but did not differ significantly among the groups (Figure 3a), implying that, overall, a similar, regular spacing is maintained between nuclei in all groups.

Nuclear number and function are prerequisites for preserving the intrinsic contractile characteristics (e.g., force per unit of CSA, termed specific force) of muscle fibers (Omairi et al., 2016). Thus, we measured the specific force of membrane‐permeabilised muscle fibers from imOX (*n* = 12), mKO (*n* = 12) and WT mice (*n* = 12 fibers from 3 animals/genotype). We did not observe any significant differences among the groups (Figure 3b). To evaluate whether there were any differences in force production across the range of fiber sizes, we plotted absolute force against fiber CSA, and again observed no difference in linear regression lines (Figure 3c).

**Figure 4:**
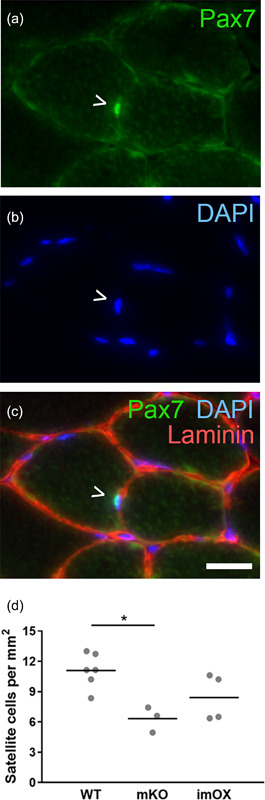
Satellite cell numbers in skeletal muscle cryosections. (a–c) Satellite cells were identified as Pax7+ nuclei residing underneath the basal lamina (green, Pax7; blue, DAPI; red, laminin). (d) Satellite cell numbers per mm^2^ of cryosection (tibialis anterior muscle). Each data point represents one animal (8 fields of view at ×20 magnification per animal). Scale bar: 20 μm

**Figure 5:**
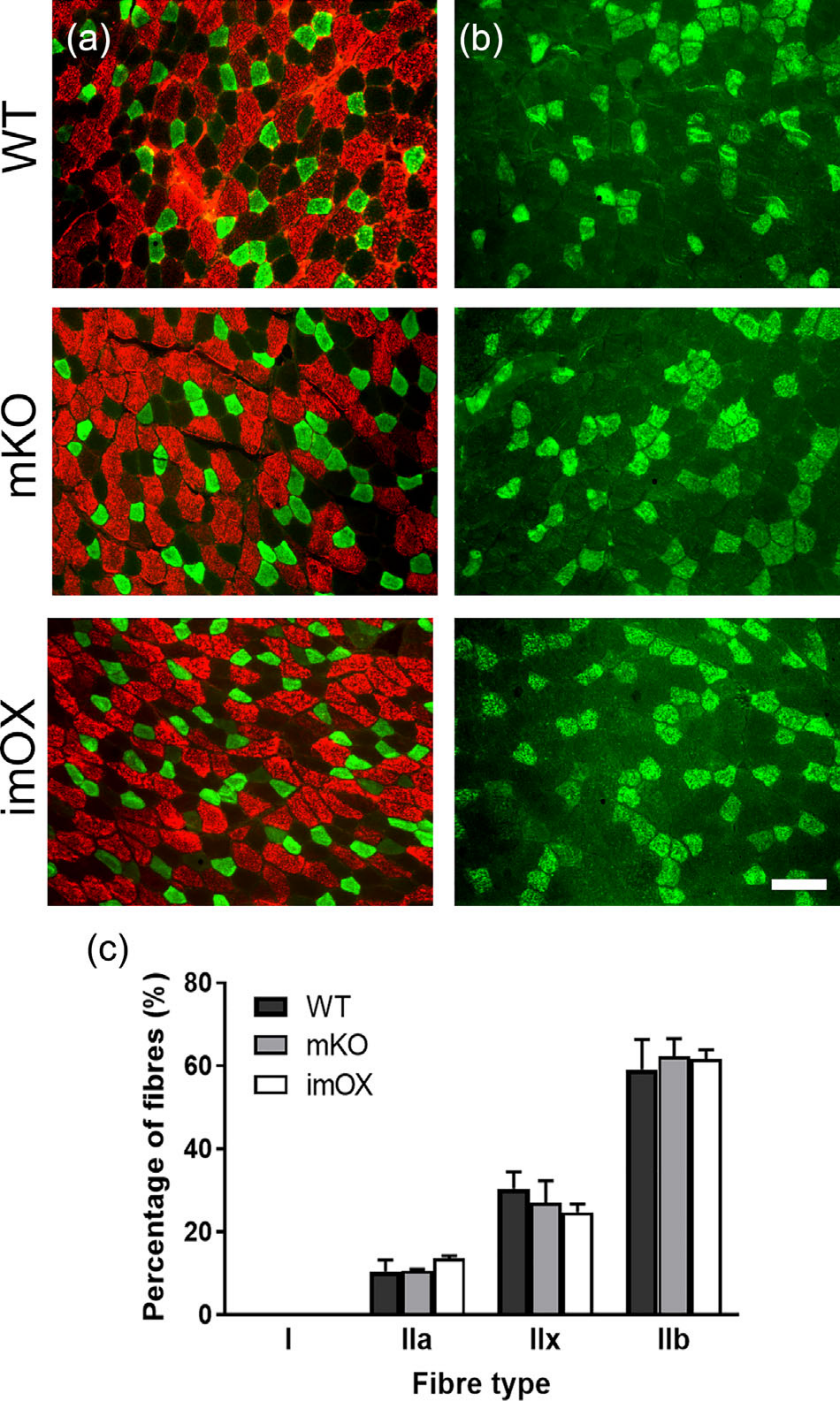
Fiber type composition in skeletal muscle cryosections. Antibodies against myosin isoforms: (a) fast/type IIa (green) and fast/type IIb (red); (b) fast type IIx (green). In addition, an antibody against type I myosin confirmed a complete absence of slow fibers (data not shown). (c) counts of each fiber type, with median and range of 3 animals per genotype. 400 + fibers counted per animal across several low magnification images. Scale bar: 50 μm

Since the addition of myonuclei to myofibers during development and hypertrophy is known to be dependent on muscle stem cells, termed satellite cells, we assessed their numbers in cryosections of skeletal muscle (Figure 4a‐c). We found that the abundance of satellite cells was reduced in mKO (*p* = 0.011), but unchanged in imOX muscles (*p* = 0.11) compared to WT mice (Figure 4d). The regulation of myonuclear number is also known to be dependent on fiber type, with slow/type I fibers displaying smaller myonuclear domains. However, we observed no differences in the relative proportions of type I, IIa, IIx, and IIb fibers among the groups (Figure 5).

## 4 DISCUSSION

In the present study, our aim was to characterise the contribution of SIRT1 to nuclear positioning and/or MND sizes in skeletal muscle fibers. We performed a series of experiments using mice where SIRT1 is inactivated (mKO) or overexpressed (imOX) specifically in skeletal muscle. Strikingly, we observed that the myonuclear number was significantly increased in imOX mice resulting in a decrease in the average MND size, whilst in mKO mice, nuclear number was decreased, resulting in increased MND size (Figures 1 and 2).

It is well known that slow‐twitch type I fibers have smaller myonuclear domains than fast‐twitch type II fibers, presumably to support the larger number of mitochondria in the former. Our results and those obtained previously suggest that the SIRT1‐dependent modulation of MND size is not dependent on fiber type or effects on mitochondrial biogenesis. This is supported by the following findings: (1) the mKO and imOX models have no alterations in the relative proportions of type I, IIa, IIx, and IIb fibers in TA muscles (Figure 5 and Svensson et al., 2017); and (2) both mKO and imOX models have no alterations in the expression of a large panel of mitochondrial markers, including those required for biogenesis (Philp et al., 2011; Svensson et al., 2017). Thus, we suggest that the effects of SIRT1 on MND size are not secondary to a shift in fiber type or oxidative capacity.

Our previous results in mice with conditional knockout or overexpression of PGC‐1α in skeletal muscle mirror the current findings related to SIRT1; increasing the levels of PGC‐1α resulted in smaller MND sizes, and ablation resulted in larger MND sizes (Ross et al., 2016). PGC‐1α is activated by SIRT1, which suggests a hierarchy of signaling elements in the determination of myonuclear number. Intriguingly, the SIRT1 imOX or mKO model do not show any alterations in PGC‐1α protein level or activation, suggesting that other proteins (for instance GCN5) are able to activate PGC‐1α in the absence of SIRT1 (Philp et al., 2011; Svensson et al., 2017). Given the complexity of the SIRT1 signaling network, this suggests that SIRT1 is able to modulate MND sizes through mechanisms that are not dependent on PGC‐1α, and that multiple factors may be involved.

How might SIRT1 activity and MND size be related? Nuclear addition in myofibers depends on a process that starts with the activation of satellite cells, the muscle resident stem cells, which then become muscle progenitors termed myoblasts. These cells then proliferate, and fuse with myofibers, resulting in the addition of new nuclei. Several lines of evidence support the fact that SIRT1 stimulates myoblast proliferation (Rathbone et al., 2009). Down‐regulating or inactivating SIRT1 in the muscles of mice deregulates the myogenic programme, delays the activation of satellite cells and impairs muscle growth and repair (Ryall et al., 2015; Tang & Rando, 2014). Our results demonstrate that satellite cell numbers are reduced in mKO mice compared with WT, a factor which might result in reduced incorporation of nuclei into muscle fibers and thus the increased MND sizes (Figures 2 and 4). However, no alterations in satellite cell numbers were observed in imOX mice, making other factors (such as the SIRT1‐dependent regulation of the myogenic programme) a likely mechanism.

The prevailing theory in the literature is that maintaining MND size is a prerequisite for preserving the intrinsic force‐generating capacity of muscle fibers (Qaisar et al., 2012). Indeed, in the myostatin null mouse, which displays hypertrophy where MND sizes are increased, perhaps resulting in nuclei being forced to extend their influence beyond their maximum capacity or physiological “ceiling.” Contractile protein density and force production are then compromised. In contrast, mice overexpressing insulin‐like growth factor 1 (IGF1) in muscle display hypertrophy with preserved MND sizes, and contractile protein density and specific force production are normal (Qaisar et al., 2012). Importantly, while the MND size increased and decreased in mKO and imOX mice, respectively, the intrinsic ability of muscle fibers to produce force was not affected. We interpret these data to suggest that the MND needs to be more extensively changed (as compared to control/healthy muscle) in order to have a detrimental impact on muscle function, and that the alterations in MND observed in this study were within a range that was appropriate for normal contractile function. It should be noted that force measurements were obtained from chemically skinned fibers, a methodology that assesses function purely at the contractile level, and excludes factors relating to, for example, calcium handling or ATP production. Therefore, one cannot rule out an effect of altered MND size on function in intact muscle. As such, it will be very interesting in further work to precisely define the point at which such change in MND negatively affects muscle functional properties.

In conclusion, our data demonstrate that SIRT1 contributes to the control of nuclear density and MND size in muscle fibers, and that there appears to be a physiological “zone,” or range of MND sizes which are compatible with normal contractile function. Considering that increasing SIRT1 activity decreased the MND volume (i.e., increased the number of nuclei), it will be of interest in future studies to investigate SIRT1 as a therapeutic target, for diseases where nuclear alteration is part of the pathophysiology, such as in muscular dystrophies.

## CONFLICTS OF INTEREST

The authors have no conflicts of interest to disclose.
